# Cross-Talk between Adiponectin and IGF-IR in Breast Cancer

**DOI:** 10.3389/fonc.2015.00157

**Published:** 2015-07-15

**Authors:** Loredana Mauro, Giuseppina Daniela Naimo, Emilia Ricchio, Maria Luisa Panno, Sebastiano Andò

**Affiliations:** ^1^Department of Pharmacy, Health and Nutritional Sciences, University of Calabria, Cosenza, Italy

**Keywords:** breast cancer, obesity, adiponectin, insulin-like growth factor-I receptor, estrogen receptor

## Abstract

Obesity is a chronic and multifactorial disorder that is reaching epidemic proportions. It is characterized by an enlarged mass of adipose tissue caused by a combination of size increase of preexisting adipocytes (hypertrophy) and *de novo* adipocyte differentiation (hyperplasia). Obesity is related to many metabolic disorders like hypertension, type 2 diabetes, metabolic syndrome, and cardiovascular disease, and it is associated with an increased risk of cancer development in different tissues including breast. Adipose tissue is now regarded as not just a storage reservoir for excess energy, but rather as an endocrine organ, secreting a large number of bioactive molecules called adipokines. Among these, adiponectin represents the most abundant adipose tissue-excreted protein, which exhibits insulin sensitizing, anti-inflammatory, and antiatherogenic properties. The serum concentrations of adiponectin are inversely correlated with body mass index. Recently, low levels of plasma adiponectin have been associated with an increased risk for obesity-related cancers and development of more aggressive phenotype, concomitantly with alterations in the bioavailability of insulin-like growth factor-I (IGF-I) and IGF-I receptor (IGF-IR) signaling pathways. In this review, we discuss the cross-talk between adiponectin/AdipoR1 and IGF-I/IGF-IR in breast cancer.

## Introduction

Breast cancer is one of the most common forms of female malignancy in the world. Many epidemiological studies suggest an important, but still controversial, role for obesity and adipose tissue mass in breast cancer risk and an association with tumor phenotypes (1). The latter event has been suggested to relay on the increased estrogen production in peripheral fat deposits, through the aromatization of androgens, secreted by the adrenal gland (2). However, on the basis of many recent studies, the contribution of obesity to the development of breast carcinoma cannot be ascribed to the increased estrogen levels only. In more recent years, it has been demonstrated that autocrine, endocrine, and paracrine-acting adipocytes-derived factors, known as adipocytokines, may contribute to the regulation of breast cancer development and progression (3). As a member of the adipocytokine family, adiponectin is synthesized and secreted almost exclusively by the adipose tissue (4), and its plasma concentration is inversely correlated with adiposity (5). Adiponectin exhibits anti-inflammatory activity, a protective effect against metabolic disorders, such as insulin resistance, fatty-acid oxidation, and insulin sensitivity.

Furthermore, recent clinical studies have implicated that the reduction of circulating adiponectin levels is a risk factor not only for type 2 diabetes and cardiovascular diseases but also for several types of cancers, including breast cancer (6, 7). However, conflicting observations have been reported on the effects elicited by adiponectin on breast cancer cell growth. It has been extensively demonstrated that adiponectin induces an anti-proliferative response in human estrogen receptor alpha (ERα)-negative breast cancer cells (8–10), while controversial data are reported in ERα-positive cells (11–16). The possible mechanisms through which adiponectin exerts anticancer effects may include activation of AMP-activated kinase and decrease of mTOR signaling (12). This addresses how a network of different signaling pathways is involved in the adiponectin-mediated effects in breast cancer, and the response to adiponectin seems to be dependent on breast cancer phenotypes.

## Obesity and Breast Carcinogenesis

Breast carcinoma represents the highest incidence of cancers affecting women, and it is the second most fatal cancer type (17, 18). Aside from the genetic predisposition, a myriad of other factors can contribute to the pathogenesis of breast cancer (19). Among the modifiable factors, obesity not only represents a serious risk for breast carcinogenesis but also positively correlated with a poor outcome (20).

**Figure 1:**
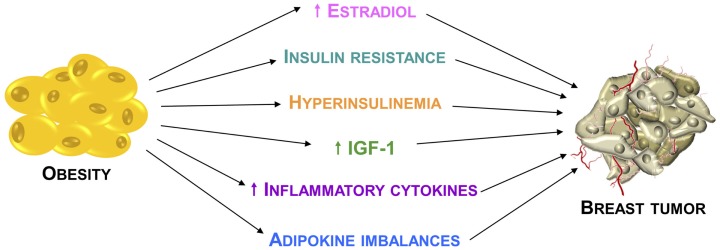
**Relationship between obesity and breast cancer**. Principal mechanisms through which the obesity condition may promote breast cancer development and progression.

It is now widely accepted that obesity may promote breast tumor through several mechanisms (Figure 1). Traditionally, the adverse effect of obesity on breast cancer prognosis has been linked to the higher estrogen levels produced, consequent to a greater aromatase activity due to the excess of adipose tissue (21). Despite the well documented relationship between obesity and estrogenic activity, it is evident that this cannot fully explain the association between body weight and breast cancer risk and prognosis.

Estrogen-independent mechanisms for which there are both experimental and epidemiological supports involve insulin resistance, hyperinsulinemia, greater bioavailability of insulin-like growth factor-I (IGF-I), which represents the more relevant obesity-related growth factor, and dysregulation of insulin-like growth factor-I receptor (IGF-IR) downstream signaling pathways (22).

Apart from these mechanisms, another important element in obesity-mediated breast carcinogenesis is represented by the interaction between tumor cells and the surrounding microenvironment, which comprises stromal cells, soluble factors, signaling molecules, and extracellular matrix that can promote tumorigenesis, and make the tumor resistant from host immunity and therapeutic response. Importantly, obesity is also characterized by multiple changes in the adipose tissue biology.

Particularly, white adipose tissue in obese individuals exhibits chronic mild inflammatory status, mostly defined by infiltration of leukocytes, including macrophages (23). For instance, stromal adipocytes directly influence breast cancer cells growth and progression through the secretion of several biologically active polypeptides known as adipokines (24, 25), which have a chemo-attractant action, causing the recruitment of macrophages. The activated macrophages release proinflammatory molecules, including TNFα, IL-1β, and IL-6. These cytokines play both local and systemic actions, contributing to insulin resistance and breast cancer tumorigenesis (26, 27).

## Adiponectin

Adiponectin, one of the most important adipokines, is produced exclusively in white adipocytes. The human gene encoding adiponectin maps to chromosome 3q27, a region associated with susceptibility for developing metabolic syndrome and type 2 diabetes in Caucasians (28). Adiponectin gene spans 16 kb and contains three exons and two introns (29). Several single nucleotide polymorphisms (SNPs) in the coding region were identified, which are associated with alterations of adiponectin function and important clinical conditions. In particular, SNPs are associated with the strengthening of adiponectin effects on insulin resistance, type 2 diabetes, obesity, dyslipidemia, and many obesity-related malignancies (30).

**Figure 2:**
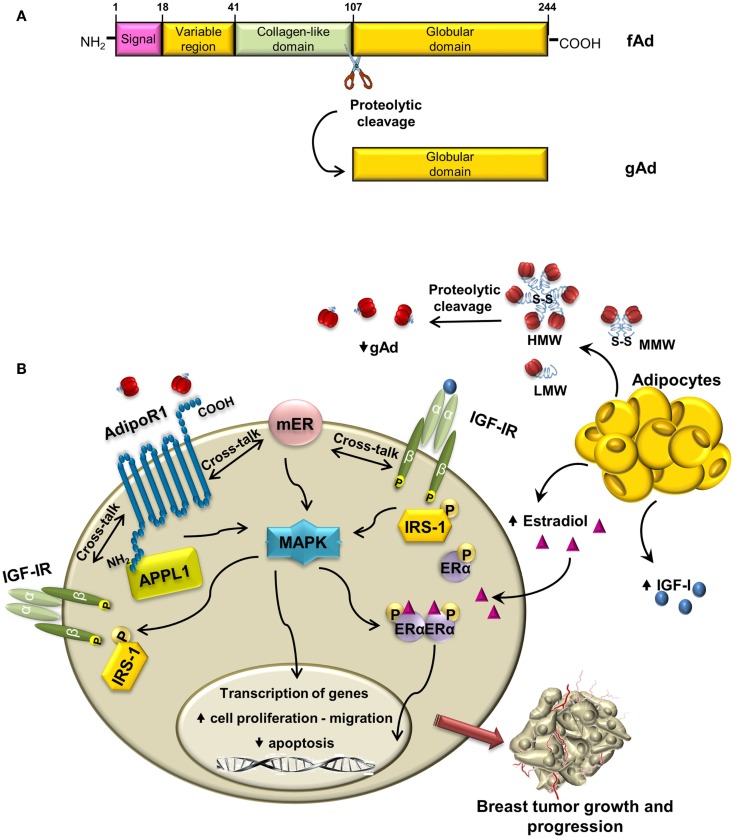
**Cross-talk between adiponectin and IGF-IR in breast cancer: overview of mechanisms**. **(A)** Schematic structure of adiponectin monomer with different domains and proteolytic cleavage site indicated. fAd and gAd indicate full length and globular adiponectin, respectively. **(B)** In ERα-positive breast cancer cells, adiponectin, produced by adipocytes, binds to AdipoR1, and establishes cross-talk with membrane ERα and with IGF-IR. The enhanced phosphorylation of MAPK may allow the activation of IGF-IR, IRS-1, and ERα, which contribute to breast tumor growth and progression.

Structurally, the molecule of human adiponectin consists of 244 amino acid residues and contains four distinct domains; at the N-terminus, there is an 18 amino acid long-signal peptide followed by a short-hypervariable region without homology to any known sequences and a collagen domain with 22 repeated motifs; C-terminal contains globular domain homologous to C1q molecule of complement cascade (Figure 2A). The C-terminal globular domain also shows homology with TNF-α trimeric cytokines family (31, 32).

Once synthesized, adiponectin undergoes posttranslational hydroxylation and glycosylation modifications (33) and before secretion it forms trimers (low-molecular weight, LMW) that oligomerize to produce hexame-rich middle molecular weight (MMW) and high-molecular weight (HMW) forms. Adiponectin circulating in plasma exists as the full-length protein (fAd) or a proteolytic cleavage fragment known as globular adiponectin (gAd) (34–36), which is probably generated by elastase digestion (37, 38) (Figure 2A).

The levels of this adipokine are abundant in human plasma, with concentrations ranging from 3 to 30 μg/ml (39), and they are about two to three times lower in male compared to female, due to the lower amounts of HMW form (40, 41). Its concentration is inversely correlated with body mass index (42). Indeed, unlike most of the other adipokines, plasma adiponectin levels are found to be lower in obese than in lean individuals (3). Moreover, low-circulating levels are found in type 2 diabetes (43), and mice lacking adiponectin develop metabolic syndrome, with insulin resistance, glucose intolerance, hyperglycemia, and hypertension (44, 45). The mechanisms responsible for the adiponectin downregulation are still unclear. It has been speculated that its reduced levels in obesity may be caused by the enhanced production of proinflammatory cytokines, in particular, by the tumor necrosis factor α (TNFα) (46). However, another potential explanation indicates a negative feedback of adiponectin on its own production and probably on the expression of its receptors during the development of obesity (47).

## Adiponectin Receptors

Adiponectin exerts many of its cellular effects through binding to two receptor isoforms, the adiponectin receptor 1 (AdipoR1) and 2 (AdipoR2) (48). AdipoR1 and AdipoR2 contain seven transmembrane domains with internal N-terminus and external C-terminus regions; thus, they are both structurally and functionally distinct from G-protein-coupled receptors (GPCR) (49). AdipoR1 presents high affinity for gAd and low affinity for the full-size ligand (50), and it is expressed ubiquitously but abundantly in skeletal muscle and endothelial cells. AdipoR2 has intermediate affinity for both forms of adiponectin and is predominantly expressed in the liver (51). Since AdipoR1 is the predominant receptor in skeletal muscle, while AdipoR2 is predominantly expressed in liver, this correlated with the fact that gAd exerts its insulin mimetic and insulin-sensitizing effect more effectively compared to fAd in skeletal muscle and *vice versa* (52).

A non-classical third potential adiponectin receptor is T-cadherin, which was also found to competitively bind only the hexameric and HMW forms of adiponectin (53–55). Various studies have suggested the involvement of T-cadherin, which plays an important role in cell adhesion and in calcium-mediated cell to cell interactions and signaling (53), in mediating functional effects of adiponectin. T-cadherin lacks an intracellular domain needed for signal transduction; thus, it has been suggested that it may function as a coreceptor by competing with AdipoR1 and AdipoR2 receptors for adiponectin binding or interfering with adiponectin signal transduction (56).

## Adiponectin Signaling Pathways

Adiponectin activates various signaling molecules when bound to its receptors. Several adiponectin receptor binding proteins have now been identified (57). Among these, the first and best characterized is the adaptor protein APPL1, containing a pleckstrin homology domain, a phosphotyrosine binding (PTB) domain, and a leucine zipper motif (57, 58). AdipoR1 and AdipoR2 interact with the PTB domain of APPL-1 through their N-terminal intracellular region, thereby inducing adiponectin actions through the sequential activation of downstream signaling. It has emerged that APPL1 plays an important role in mediating many of adiponectin’s effects, including metabolic, antiinflammation, antiatherogenic, and cytoprotection responses (59, 60).

Adiponectin exerts its effects through the activation of AMPK, mTOR, PI3K/Akt, MAPK, PPAR-α, STAT3, and NF-kB (47, 59).

Most of its effects are mediated through the cellular energy sensor AMPK (61, 62), which promotes glucose utilization that results in an increased fatty-acid oxidation, increased glucose uptake at the skeletal muscle level, and reduced gluconeogenesis in the liver (47). In addition, adiponectin exerts its insulin-sensitizing effects through the activation of PPAR-α, thereby enhancing fatty-acid combustion and energy consumption, leading to a tissue decrease content of triglycerides in the liver and skeletal muscle, and improving insulin sensitivity *in vivo* (63).

## Adiponectin and Breast Cancer

Adiponectin is emerging as an important factor in carcinogenesis. Many clinical investigations suggested that low-adiponectin concentrations are associated with an increased risk for obesity-related cancer, such as prostate, colon, endometrial, and breast cancer (47). Epidemiological studies address that, in women with low-circulating adiponectin levels, breast tumors may present a more aggressive phenotype, exemplified by large tumor size, high-histological grade, estrogen receptor negativity, and increased angiogenesis and metastasis (6). Recently, through a transcriptomic profiling, Merdad and co-workers evidenced a downregulation of adiponectin and other molecules involved in lipid metabolism in surgically resected breast tumors patients. The results address how some ethnic groups are more susceptible to breast cancer occurrence (64).

It has been well documented that the pathogenesis of mammary cancer is not only dependent on genetic alterations but also largely on the interactions between malignant cells and components of the breast microenvironment, which exerts an important influence on the phenotype of the neoplastic cells and on tumor progression. Microenvironmental molecules, such as chemokines, cytokines, and growth factors, could influence tumor progression by three major, non-mutually exclusive, mechanisms. The first is by further increasing the genetic instability of tumor cells. The second is by inducing signaling cascades in tumor cells via tumor-associated receptors thereby controlling gene expression in these cells. The third mechanism is by exerting selective pressures on the cells (24, 65).

Thus, the malignant cell phenotype is regulated not only by autonomous signals originating from cancer cells but also by the effects of the surrounding stromal cells, which influence mammary epithelial cell growth and differentiation (24). The close association between mammary epithelial cells and adipocytes may favor a more direct action of adipokines on such tissue (11).

However, the role of this adipokine on breast tumorigenesis is still unclear, and it seems to be dependent on cell types. Low-adiponectin doses mediate an anti-proliferative response in ERα-negative breast cancer cells through the regulation of genes involved in cell cycle, such as p53, Bax, Bcl-2, c-myc, and Cyclin D1 (66). In addition, in these cells, the adipokine is able to activate the AMPK, which in turn inhibits mTOR, through tuberous sclerosis complex 2 (TSC2), thus counteracting carcinogenesis (67, 68). Moreover, activated AMPK plays a crucial role in the regulation of growth arrest and apoptosis by stimulating p21 and p53 (69). On the other hand, it is emerging that adiponectin, at low concentrations, increases proliferation in ERα-positive breast cancer cells (15, 16). This occurs through the activation of ERα at both genomic and non-genomic levels (70), together with the positive regulation of cyclin D1 expression (71).

On the basis of these observations, adiponectin may represent a promising diagnostic and prognostic biomarker to identify high-susceptibility individuals for developing obesity-related tumors. Recently, it has developed an adiponectin-based short peptide, ADP 355, acting as AdipoR1 agonist, and able to modulate several signaling pathways (AMPK, Akt, STAT3, and ERK1/2) in a manner similar to gAd. In breast cancer cells, ADP 355 reproduces the adiponectin-induced anti-proliferative activity both *in vitro* and *in vivo* (3, 72, 73).

## IGF-IR in Breast Cancer

Insulin-like growth factor-I receptor is an evolutionary conserved, ubiquitous transmembrane tyrosine kinase structurally similar to the insulin receptor. IGF-IR regulates different biological processes, such as proliferation, survival, differentiation, transformation, cell-substrate, and cell–cell interactions (74–79). More recently, it has been highlighted that the role of the IGF-IR in the development and maintenance of the cancer stem cells, in epithelial–mesenchymal transition, and in the regulation of the tumor microenvironment (80). The first evidence that IGF-IR is critical in tumorigenesis was provided by the observation that constitutive overexpression of IGF-IR induced the transformed phenotype in cultured cells (81, 82).

It is now quite evident that IGF-IR plays a multifaceted and complex role in the development and progression of a malignant disease. Numerous clinical and experimental data indicated that IGF-IR is overexpressed in several subtypes of breast cancer (83), and many conditions lead to the activation of IGF-IR tyrosine kinase activity (84), allowing interaction with its main substrates, such as insulin receptor substrates (IRS) and the Src-homology-2-containing protein SH2 (SHC) (74). Once phosphorylated, these proteins act as docking molecules that bind to and activate cellular kinases, initiating different downstream signaling pathways. In this regard, IGF-IR induces activation of Ras/raf/MAPK and PI3K/Akt signaling, which alter the expression of genes involved in cell proliferation and survival (85), thus contributing to breast carcinogenesis.

In hormone-dependent breast cancer cells, the IGF-IR and ERα are co-expressed and IGF-I acts in synergy with estradiol to stimulate proliferation (74). Indeed, estradiol up-regulates IGF-IR mRNA and protein levels as well as its tyrosine phosphorylation (73, 74, 86). Furthermore, estradiol significantly stimulates the expression of IRS-1 in ERα-positive cells (87).

Elevated expression of IGF-IR or IRS-1 appears to increase drug and radio resistance of breast cancer cells and favor cancer recurrence in patients (88, 89). In addition to promote cell growth, IGF-IR counteracts apoptosis in ERα-positive cells, activating the IRS-1/PI3K pathway (74, 89). For these reasons, IGF axis has been validated for developing targeted therapy for cancer prevention and treatment (80, 90, 91).

## Adiponectin and IGF-IR Interaction in Breast Cancer

Insulin-like growth factors and IGF-IR are involved in the metabolic cellular response to adiponectin in many cell types (63). It has been widely demonstrated that IGF-IR increases growth and survival of neoplastic cells, altering the expression of specific genes regulated through the activation of Akt and MAPK pathways (92).

Emerging evidences address the existence of a cross-talk between adiponectin/AdipoR1 and IGF-IR in breast cancer. It has been previously demonstrated that adiponectin increases IGF-IR-β subunit tyrosine phosphorylation and the downstream MAPK activation in granulosa cells (93). More recently, it has been shown that low concentrations of adiponectin rapidly increase IGF-IR phosphorylation in ERα-positive breast cancer cells, concomitantly with ligand-independent activation of ERα (70). It is worth to note that in MCF-7 cells, upon adiponectin treatment, knockdown of ERα reduces IGF-IR phosphorylation, whereas a specific siRNA for IGF-IR prevents adiponectin-induced ERα transactivation (70).

Moreover, IGF-IR takes part in the formation of a protein complex involved in the induction of ERα-positive breast cancer cell growth. Indeed, adiponectin enhances the coimmunoprecipitation of AdipoR1, APPL1, ERα, IGF-IR, and c-Src in MCF-7 cells. The formation of this multiprotein complex leads, via c-Src, to MAPK activation, which is abrogated in the presence of specific RNA silencers targeting ERα or IGF-IR (70). On the other hand, in ERα-negative MDA-MB-231 cells adiponectin is no longer able to induce MAPK phosphorylation, but it increases activation of AMPK, which mediates anti-proliferative effect in these cells (70, 71). In this concern, it has been well documented how in breast cancer cells the adiponectin-induced activation of AMPK is concomitant with inactivation of MAPK (12).

Interestingly, our unpublished data demonstrate that adiponectin enhances phosphorylation and protein expression of IRS-1 in ERα-positive breast cancer cells, thus amplifying the IGF-I/IGF-IR growth signaling. All these data address the existence of a possible cross-talk between Adiponectin/AdipoR1, ERα, and IGF-IR involved in the positive regulation of ERα-positive breast cancer cell growth. These data are supported by other papers showing that adiponectin, via its adapter protein APPL1, is able to induce phosphorylation of IRS-1, which mediates the effect of activated IR/IGF-IR in normal and tumoral cells (63, 94).

Our preliminary results surprisingly show that adiponectin at low concentrations is able to potentiate IGF-I-induced anchorage-independent growth in ERα-positive breast cancer cells. In contrast, adiponectin is able to elicit opposite action in antagonizing the stimulatory effects induced by IGF-I in ERα-negative breast cancer cells. In the same vein, wound healing assays evidence that in ERα-positive breast cancer cells, adiponectin, and IGF-I enhance cell motility and the co-treatment results in an additive effect, addressing how adiponectin may synergize with IGF-IR signaling. On the contrary, in ERα-negative breast cancer cells adiponectin counteracts the IGF-I-induced cell migration (manuscript in preparation). These results well fit with recent findings demonstrating that adiponectin is able to regulate migration and invasion in different breast cancer cells (95–97).

## Conclusion

Adiponectin is emerging as a crucial adipokine involved in breast carcinogenesis in women with obesity. In ERα-positive breast cancer cells, the interaction of adiponectin with its specific receptor induces the activation of multiple pathways, through the interplay between ERα and IGF-IR. This leads to (i) increased activation of MAPK and upregulation of genes involved in proliferation and inhibition of apoptosis, (ii) induction of cell migration (Figure 2B). On the basis of these findings, we may conclude that adiponectin differently modulates IGF-I stimulatory effect in breast cancer cells in relationship to ERα status. Indeed, the antagonistic effects exerted by adiponectin on IGF-IR signaling are evident only in ERα-negative breast cancer cells. Thus, only in the latter circumstance, adiponectin sounds to be exploited in novel therapeutic strategies for breast cancer treatment.

## Conflict of Interest Statement

The authors declare that the research was conducted in the absence of any commercial or financial relationships that could be construed as a potential conflict of interest.
